# Differential predictors of expressed emotion toward individuals with schizophrenia between families and halfway houses

**DOI:** 10.3389/fpsyt.2024.1322809

**Published:** 2024-03-14

**Authors:** Panagiotis Ferentinos, Stamatina Douki, Eleni Kourkouni, Dimitra Dragoumi, Nikolaos Smyrnis, Athanassios Douzenis

**Affiliations:** ^1^ 2nd Department of Psychiatry, “Attikon” University General Hospital, National and Kapodistrian University of Athens, Athens, Greece; ^2^ Department of Psychiatry, “Evangelismos” General Hospital, Athens, Greece; ^3^ Center for Clinical Epidemiology and Outcomes Research, Athens, Greece

**Keywords:** criticism, differential predictors, emotional overinvolvement, expressed emotion, families, halfway houses, schizophrenia

## Abstract

**Background:**

This study investigated patient- and caregiver-related predictors of expressed emotion (EE) toward individuals with schizophrenia in families and halfway houses and yet understudied differential effects across settings.

**Methods:**

We included 40 individuals with schizophrenia living with their families (“outpatients”) and 40 “inpatients” in halfway houses and recorded the EE of 56 parents or 22 psychiatric nurses, respectively, through Five Minutes Speech Sample. Each outpatient was rated by one to two parents; each inpatient was rated by two to five nurses. As EE ratings had a multilevel structure, EE predictors were investigated in backward stepwise generalized linear mixed models using the “buildmer” R package. We first fitted models including either caregiver- or patient-related predictors in each setting and finally included both types of predictors. Setting-specific patient-related effects were investigated in interaction analyses. Adjustment for multiple tests identified the most robust associations.

**Results:**

In multivariate models including either caregiver- or patient-related predictors, nurses’ higher age, shorter work experience and lower inpatients’ negative symptoms robustly predicted higher emotional overinvolvement (EOI). In the final models including both types of predictors, nurses robustly displayed lower EOI (i.e., reduced concern and disengagement) toward inpatients with higher negative symptoms. Several other features were nominally associated with criticism and EOI in each setting. However, no feature robustly predicted criticism in inpatients and criticism/EOI in outpatients after adjustment for multiple tests. In interaction analyses, higher negative symptoms differentially predicted lower EOI in nurses only.

**Conclusion:**

Our findings suggest setting-specific pathogenetic pathways of EOI and might help customize psychoeducational interventions to staff in halfway houses.

## Introduction

Schizophrenia is a chronic psychiatric disorder, affecting less than 1% of the general population and presents with positive symptoms (delusions and hallucinations), disorganization symptoms (disorganized thought, speech, and behavior), negative symptoms (avolition, poverty of speech, and flattened affect), and often cognitive impairment (1). It typically starts in early adulthood and is often associated with social/occupational decline (2) and early mortality mainly due to high rates of comorbid medical conditions (3). Predictors of worse prognosis include reduced compliance to treatment, substance misuse, poor premorbid adjustment and a critical family environment (4).

Expressed emotion (EE) is a construct introduced in the 1950s to study the effect of intrafamilial emotional communication on the course of schizophrenia (5, 6). It refers mainly to criticism and emotional overinvolvement (EOI) from family members toward the individual with schizophrenia but also encompasses hostility (rejection) as well as positive aspects, i.e., warmth (i.e., concern for patients) and positive comments; hostility is highly correlated with criticism while warmth is negatively correlated with criticism and positively with EOI (7). EE is traditionally assessed not only with the Camberwell Family Interview (CFI), but also with the less time-consuming Five Minutes Speech Sample, (FMSS) measuring only criticism and EOI, and several self-report scales (8). Meta-analyses of various prospective studies have documented the negative impact of high EE, particularly high criticism, on clinical outcomes in family settings while high warmth acts protectively (9–11). Consequently, family psychoeducational interventions have proved a useful adjunct to antipsychotic medication in the management of schizophrenia (12, 13).

More recently, research on EE in schizophrenia has extended to the staff (usually psychiatric nurses) of psychosocial rehabilitation services, such as supported housing facilities in the community. Among them, halfway houses (transitional hostels) stand midway between a recent psychiatric hospitalization and independent community living, providing for a limited time frame housing away from a dysfunctional family, education, and support toward social reintegration. Rates of high EE in staff are often lower than in family relatives and almost always arise from criticism rather than EOI, since staff may emotionally invest less in patients (14). Staff EE has also been associated with patient outcomes though weakly, inconsistently, and in much fewer prospective studies (15, 16).

Given the importance of EE for clinical outcomes, various studies have investigated patient- and caregiver-related EE predictors in families or halfway houses (14, 17). However, it is unknown whether predictors of criticism and EOI are common or different across these settings since studies including individuals from both are unfortunately absent. Indirect comparisons of findings from a highly heterogeneous literature can be biased, since studies often include different sets of predictors in their models and use different EE measures and scoring algorithms. For example, FMSS and CFI have modest concordance rates, with FMSS considered less sensitive in identifying high-EE individuals (8). Finally, heterogeneity of sample characteristics may often confound associations, e.g., hostel residents are often more chronic, of lower socioeconomic and educational status than individuals living in families, but usually required to be remitted before admission, while nurses are often younger and more educated than parents (18).

This study included individuals with schizophrenia living in halfway houses or with their families and recorded the EE of the caring staff or parents, respectively. It aimed to identify (a) staff- or parent-related and patient-related EE predictors in the two settings; (b) differential predictors of EE among staff and parents. We hypothesized that these predictors would be positively or negatively associated with EE outcomes and that patterns would be similar across settings. We first identified caregiver-related EE predictors in families and hostels. Then, we used the same set of patient-related characteristics (clinicodemographic variables, psychopathology, and perceived criticism) to identify shared and distinct EE predictors in the two settings through interaction analyses. Finally, we combined caregiver- and patient-related EE predictors in each setting.

## Materials and methods

### Participants

A convenient sample of a total of 80 individuals of both sexes diagnosed with schizophrenia using Structured Clinical Interview for DSM-5 Disorders, Clinician Version (SCID-5-CV) (19), aged 18–65 years, was recruited from February 2018 to February 2020; 40 “inpatients” lived in four transitional halfway houses (psychiatric hostels) for at least 3 months and 40 “outpatients” lived with their families and were followed-up in two general hospital outpatient clinics. All patients had to be on antipsychotic medication, free of relapse, and in no need for psychiatric hospitalization during the last 3 months. Exclusion criteria for inpatients’ admission into the hostels were intellectual disability, history of alcoholism or drug use in the last 6 months, and current severe medical conditions (e.g., neurological degenerative diseases and brain lesions); these were also applied in recruiting both inpatients and outpatients. Clinicodemographic characteristics were recorded.

In addition, all 22 nurses working in the four halfway houses and caring for the 40 inpatients and 56 parents of the 40 outpatients also participated in the study as raters of their attitudes and feelings (i.e., EE) toward patients. An additional exclusion criterion for raters was lifetime diagnosis of psychotic disorder.

Both patients and raters were ensured about the anonymity and confidentiality of all data requested and provided written informed consent before participation in the study. The research protocol followed the principles of the Helsinki Declaration and was approved by the Research Ethics Committees of all mental health facilities involved (National and Kapodistrian University of Athens, “Attikon” University General Hospital code 1718011361, 04-12-2017; “Evangelismos” General Hospital code 339, 21-12-2017; “Sotiria” General Hospital code 26507, 08-02-2018; and Psychiatric Hospital “Dromokaiteion” code 7355/805905, 21-05-2019).

### Measurements

Patients of both groups went through the following evaluations:

**- Brief Psychiatric Rating Scale (BPRS**): BPRS originally included 16 interviewer-rated items assessing the intensity of symptoms of schizophrenia (20). The most commonly used 18-item version (with the addition of excitement and disorientation in 1966) has a five-factor structure, namely, Thinking disorder, Withdrawal, Anxiety–Depression, Hostility–Suspicion, and Activity factors (21).
**- Perceived Criticism (PC):** The PC instrument was introduced to measure perceived criticism in a sample of depressed patients and their spouses (22). It consists of only one self-rated question rated on a 10-point Likert scale: “How critical do you feel hostel nurses/your parents have been of you overall in the last month?”.

Staff nurses caring for inpatients underwent the following assessment:

**- Maslach Burnout Inventory (MBI)**: The scale of professional burnout was designed by Maslach and Jackson in 1981 and amended in 1996 (23, 24). It includes 22 self-evaluation items scored 0–6 and explores the feelings and attitudes of professionals in their work. The scale consists of three subscales measuring Emotional Exhaustion (nine items), Depersonalization (five items), and Personal Achievements (eight items); higher scores in the first two subscales and lower ones in the third suggest higher burnout.

The parents of outpatients living with their families were evaluated as follows:

**- Family Burden Scale (FBS)**: The FBS was designed by Madianos and Economou in 1993 and was amended to its final form in 2004 (25). It is a structured interview for the relatives of individuals with schizophrenia and explores the burden of mental illness on them in the last 6 months. The scale consists of four subscales with 23 questions in total rated 0–2: Financial Burden (five items), Impact on Daily Activities and Social Life (eight items), Aggressive Behavior (four items), and Impact on Health (six items). The first three subscales indicate the objective burden, while the fourth indicates the subjective burden. The total score ranges from 0 to 46.

Finally, both staff nurses and outpatients’ parents participated in the following procedures:

**- Five Minutes Speech Sample (FMSS):** The FMSS (26) is a tool for measuring EE. In relation to the CFI, the standard assessment tool of EE, the FMSS is easier to use, needs far less time to administer, and requires shorter training of the interviewer. It can also be used even when the investigator does not know the patient very well. Each rater (care-provider or family member) is asked to talk continuously for 5 min about each patient (in his/her absence) and the interview is audiotaped. All recorded 5-min interviews are then scored according to specific rules based on the assessment of (a) the initial statement (content and voice tone), (b) the quality of the patient–rater relationship, (c) the number of negative or positive comments, and (d) the display or report of specific behaviors during the interview (see **Supplementary Methods** for details on scoring). Every 5-min speech sample is eventually characterized as high, borderline, or low on Criticism and EOI; combined classifications also arise. Apart from categorical ratings, the number of critical comments and the number of positive attitude statements were used as EE outcomes in this study. FMSS interviews were scored by a trained author (S.D.) and acceptable inter-rater agreement with another trained author (P.F.) was recorded in 20 interviews (criticism, EOI kappa = 0.89; critical comments ICC = 0.91; positive attitude statements ICC = 0.90).

### Statistical analysis

The distribution of all variables was explored with descriptive statistics. Normality was checked with the Shapiro–Wilk test and graphically with histograms and QQ-plots. Reliability (internal consistency) of the returned questionnaires was evaluated with Cronbach’s alpha. Differences in characteristics of participants (patients and nurses or parents) and EE outcomes between the two groups were evaluated with chi-square, Fisher’s exact, or Mann–Whitney tests, as appropriate. As frequencies of low FMSS-Criticism/EOI categories were very small, they were collapsed with borderline FMSS-Criticism/EOI categories in downstream univariate and multivariate analyses.

EE ratings derived from FMSS interviews were a multi-level dataset of observations. In particular, each inpatient received ratings from various nurses and each nurse rated several inpatients (“crossed levels”) while parents’ ratings were nested within outpatients. Mixed models with crossed effects did not converge on most occasions. Therefore, nurse-related characteristics associated with nurses’ EE were evaluated in inpatients using simpler mixed models with ratings nested within nurses while parent-related characteristics associated with parents’ EE were evaluated in outpatients using mixed models with ratings nested within patients. Finally, patient-related characteristics (including BPRS subscales) associated with raters’ (nurses’ or parents’) EE were evaluated in mixed models in each patient group with ratings nested within patients. Depending on the EE outcome evaluated (binary or count), generalized linear logistic (FMSS-Criticism, FMSS-EOI) or negative binomial (FMSS critical comments)/Poisson (FMSS positive attitude statements) mixed models were fitted. Univariate models were first assessed. Finally, for each outcome, the most parsimonious model with the best fit was selected with backward stepwise elimination using the “buildmer” R package based on Akaike’s Information Criterion (AIC). In a last step, rater- and patient-related predictors from multivariate stepwise models were combined to predict each EE outcome.

Finally, interactions of patient-related predictors with patient group for all EE outcomes were tested one at a time in the total sample in modified multivariate mixed models hosting all predictors included in stepwise models in either patient group. Significant interactions were plotted.

Statistical analysis was conducted in STATA MP v17 and R 4.1.2. Given the exploratory nature of the study, the level of statistical significance was set to 5%. However, to avoid type I error inflation from multiple tests (27 univariate tests in inpatients and 29 in outpatients for each of the four EE outcomes, totaling 224 tests), a strict adjusted cutoff of *p* < 0.00022 was applied to identify the most robust effects. Since outcomes for each EE component were highly correlated, a more relaxed cutoff was also considered for each group (inpatients *p* < 0.00093; outpatients *p* < 0.00086).

## Results

### Sample descriptives, univariate comparisons, and correlations

#### Patients (inpatients–outpatients)

Clinicodemographic characteristics of the two patient groups are presented in **Supplementary Table 1**. Inpatients were older (*p* = 0.001) and less well educated (*p* = 0.002) than outpatients and had a longer disease duration (*p* = 0.047) as well as more hospitalizations (*p* = 0.012).

All patients’ questionnaires had adequate reliability (Cronbach’s *α* > 0.7) (**Supplementary Table 2**). Outpatients had significantly higher scores in BPRS Withdrawal (*p* = 0.015), BPRS Total (*p* = 0.027), and PC (*p* = 0.001) than inpatients (**Supplementary Table 1**). However, both patient groups were overall remitted/mildly ill (27).

#### Raters (nurses–parents)

Demographics for nurses and parents are presented in **Supplementary Table 3**. Nurses were significantly younger (*p* < 0.001) and better educated (*p* = 0.001) than parents. Seven (12.5%) parents had a lifetime psychiatric history of depression. All raters’ questionnaires (MBI and FBS) had adequate reliability (**Supplementary Table 2**). Overall, nurses scored moderate on MBI Personal Achievements and low on other MBI subscales (23) while parents scored low on FBS total, with 12 (21.4%) scoring >24 (25).

#### EE ratings

Each of the 22 nurses was involved in 1–12 EE ratings (FMSS interviews); each of the 40 inpatients was rated by two to five nurses. A total of 155 ratings were performed by nurses on inpatients. All 56 parents rated their offspring; each of the 40 outpatients was rated by one or both parents.

A comparison of primary EE outcomes among groups is displayed in **Table 1**. Parents scored high on FMSS-Criticism and FMSS-EOI more often than nurses, but the difference was not significant; yet, they made fewer critical comments than nurses (also non-significant) but made significantly more positive attitude statements (*p* = 0.003). Finally, a marginally significant (*p* = 0.049) difference in the distribution of combined EE categories but not binary EE categories (high vs. low EE) was detected among groups.

**Table 1:** Comparison of expressed emotion (EE) outcomes between nurses/inpatients and parents/outpatients.

Five minutes speech sample (FMSS) outcomes	Nurses^a^	Parents^b^	*p*-value
**Criticism**			0.310^†^
High	84 (54.2%)	33 (58.9%)	
Borderline	65 (41.9%)	23 (41.1%)	
Low	6 (3.9%)	0 (0.0%)	
**Critical comments**	0 (0–3)	0 (0–2)	0.446
**Emotional overinvolvement (EOI)**			0.940**^††^**
High	72 (46.5%)	27 (48.2%)	
Borderline	65 (41.9%)	22 (39.3%)	
Low	18 (11.6%)	7 (12.5%)	
**Positive attitude statements**	0 (0–1)	1 (0–1)	**0.003**
**EE categories (*n* = 7)**			**0.049** ^§^
High critical	54 (34.8%)	17 (30.4%)	
High EOI	42 (27.1%)	11 (19.6%)	
High critical+EOI	30 (19.4%)	16 (28.6%)	
Borderline critical	1 (0.6%)	4 (7.1%)	
Borderline EOI	0 (0.0%)	0 (0.0%)	
Borderline critical+EOI	28 (18.1%)	8 (14.3%)	
Low critical+EOI	0 (0.0%)	0 (0.0%)	
**EE categories (*n* = 2)**			0.659
High EE	126 (81.3%)	44 (78.6%)	
Low EE	29 (18.7%)	12 (21.4%)	

N(%) or median (IQR: 25th to 75th percentiles) are presented.

Chi-square, Fisher’s exact or Mann–Whitney tests were used as appropriate.

a Data come from 155 nurses’ ratings (FMSS interviews); each of the 22 nurses was involved in 1–12 ratings; each of the 40 inpatients was rated by two to five nurses.

b All 56 parents were involved in an FMSS interview for their offspring; each of the 40 outpatients was rated by one or both parents.

^†^ p = 0.541 for high vs. borderline/low FMSS-Criticism.

^††^ p = 0.821 for high vs. borderline/low FMSS-EOI.

^§^ p = 0.405 for four FMSS categories (high critical, high EOI, high critical + EOI, and borderline/low critical + EOI).

Boldface values denote p < 0.05.

### Rater-related predictors of EE outcomes in the two groups

Univariate mixed models with rater-related predictors for all EE outcomes were first fitted in each patient group (**Supplementary Table 4A**), followed by multivariate stepwise mixed models. In inpatients, higher criticism (in any criticism outcome, FMSS-Criticism or FMSS critical comments) was significantly predicted by higher nurses’ age; higher EOI (in any EOI outcome, FMSS-EOI or FMSS positive attitude statements) was significantly predicted by nurses’ female gender, higher age, shorter work experience, and lower MBI Personal Achievements (**Table 2A**). In outpatients, higher criticism was significantly predicted by higher parents’ FBS Aggressive Behavior; higher EOI was significantly predicted by parents’ female gender, higher age, and higher FBS Impact on Activities/Social Life (**Table 2B**). Only the effects of nurses’ higher age and shorter work experience on EOI survived our strict adjusted cutoff.

**Table 2:** Multivariate stepwise models of expressed emotion outcomes in (A) hostels (40 inpatients, 22 nurses, and 155 FMSS ratings) and (B) families (40 outpatients, 56 parents, and 56 FMSS ratings), including caregiver- or patient-related predictors only (upper line of each cell) or both (lower line of each cell).

(a) Hostels	FMSS-Criticism (OR, *p*)	FMSS critical comments (IRR, *p*)	FMSS-EOI (OR, *p*)	FMSS positive attitude statements (IRR, *p*)
Nurse Predictors	Logit, Nurses	NB, Nurses	Logit, Nurses	Poisson, Nurses
Gender (female vs. male)			**4.38, 0.031** 2.93, 0.064	
Age (years)	**1.05, 0.048** **1.10, 0.008**		1.09, 0.077 1.06, 0.180	1.10, 0.0002** **1.05, 0.018**
Family status (married vs. single)				
Education (higher vs. secondary)				
Work experience (Ref. <5 years)			5–11 years: 0.15, 0.063 **0.17, 0.038** >11 years: **0.08, 0.014** **0.10, 0.012**	5–11 years: **0.25, 0.010** **0.40, 0.033** >11 years: 0.12, 5.6E-05** **0.26, 0.003**
MBI emotional exhaustion			1.06, 0.110 1.02, 0.538	1.03, 0.093 1.00, 0.889
MBI personal achievements				**0.93, 0.036** 0.97, 0.297
MBI depersonalization				
Patient Predictors	Logit, Patients	NB, Patients	Logit, Patients	Poisson, Patients
Gender (female vs. male)	0.42, 0.115 0.34, 0.051			1.48, 0.097 1.41, 0.158
Age (years)			**1.09, 0.002** **1.08, 0.008**	
Family status (ever married vs. single)				
Education (university or higher vs. lower)				
Employment (Ref. employed)		Unemployed: **0.37, 0.010** **0.37, 0.010** Pensioner: 1.45, 0.571 1.45, 0.571		Unemployed: 6.61, 0.079 4.07, 0.204 Pensioner: 9.41, 0.051 5.97, 0.128
Smoking				
Disease duration			**0.95, 0.036** **0.94, 0.036**	
No. of previous hospitalizations	1.14, 0.175 1.14, 0.162		1.17, 0.075 1.14, 0.137	
History of violent behavior				
History of suicide attempts				**2.46, 0.033** 1.79, 0.203
BPRS thinking disorder				
BPRS withdrawal	1.11, 0.193 1.12, 0.158		0.64, 9.4E-06** 0.70, 0.00052*	0.77, 0.00073* **0.79, 0.005**
BPRS anxiety/depression				
BPRS hostility/suspicion	1.20, 0.259 1.28, 0.119		0.81, 0.088 0.79, 0.065	**0.78, 0.011** **0.77, 0.011**
BPRS activity				1.21, 0.073 1.14, 0.239
Perceived criticism	**1.34, 0.016** **1.36, 0.010**	1.15, 0.051 1.15, 0.051		0.90, 0.070 0.89, 0.059
(b) Families	FMSS-Criticism (OR, *p*)	FMSS critical comments (IRR, *p*)	FMSS-EOI (OR, *p*)	FMSS positive attitude statements (IRR, *p*)
Parent Predictors	Logit, Patients	NB, Patients	Logit, Patients	Poisson, Patients
Relation/Gender (mother vs. father)			**7.89, 0.006** 24.69, 0.161	
Age (years)			**1.13, 0.009** 1.19, 0.182	
Education (Ref. primary school)				
Currently employed				
Psychiatric history				
FBS financial burden				
FBS impact on activities/social life			**1.29, 0.022** 1.59, 0.093	
FBS aggressive behavior	3.08, 0.051 3.08, 0.051	**1.90, 0.003** **1.89, 0.002**	0.56, 0.076 0.35, 0.186	
FBS impact on health			0.80, 0.059 0.70, 0.203	
Patient predictors	Logit, Patients	NB, Patients	Logit, Patients	Poisson, Patients
Gender (female vs. male)			2.25, 0.153 7.02, 0.185	**2.01, 0.034** **2.01, 0.034**
Age (years)				
Family status (ever married vs. single)		**6.46, 0.005** **5.82, 0.002**		
Education (university or higher vs. lower)		2.77, 0.130 **3.76, 0.036**		
Employment (Ref. employed)				
Smoking				1.60, 0.162 1.60, 0.162
Disease duration				
No. of previous hospitalizations				0.87, 0.050 0.87, 0.050
History of violent behavior				
History of suicide attempts		6.77, 0.052 **6.28, 0.018**		
BPRS thinking disorder				
BPRS withdrawal		1.15, 0.060 1.06, 0.390		
BPRS anxiety/depression				
BPRS hostility/suspicion			0.81, 0.100 0.67, 0.207	
BPRS activity				1.14, 0.064 1.14, 0.064
Perceived criticism				

In each cell, upper line= caregiver- or patient-related predictors only, lower line= both caregiver- and patient-related predictors.

BPRS, Brief Psychiatric Rating Scale; FBS, Family Burden Scale; FMSS, Five Minutes Speech Sample; MBI, Maslach Burnout Inventory.

Logit = Binary Logistic Generalized Linear Mixed Model; NB = Negative Binomial Generalized Linear Mixed Model; Poisson = Poisson Generalized Linear Mixed Model.

Nurses = ratings were nested within nurses; Patients = ratings were nested within patients.

In models including both caregiver and patient predictors, ratings were nested within patients.

OR > 1 and IRR > 1 denote positive associations.

Boldface values denote p < 0.05; ** p < 0.00022 (strict adjusted cutoff); * inpatients p < 0.00093, outpatients p < 0.00086 (relaxed adjusted cutoff).

### Patient-related predictors of EE outcomes in the two groups

Univariate mixed models with patient-related predictors for all EE outcomes were first fitted in each patient group (**Supplementary Table 4B**), followed by multivariate stepwise mixed models (**Tables 2A, B**). In inpatients, higher criticism was significantly predicted by being employed vs. unemployed and higher PC; higher EOI was significantly predicted by higher age, lower disease duration, history of suicide attempts, and lower BPRS Withdrawal and Hostility/Suspicion. In outpatients, higher criticism was significantly predicted only by being ever married; higher EOI was significantly predicted only by female gender. Only the effect of inpatients’ BPRS Withdrawal on EOI survived our strict adjusted cutoff.

### Interactions of patient-related predictors with group for EE outcomes

**Figure 1:**
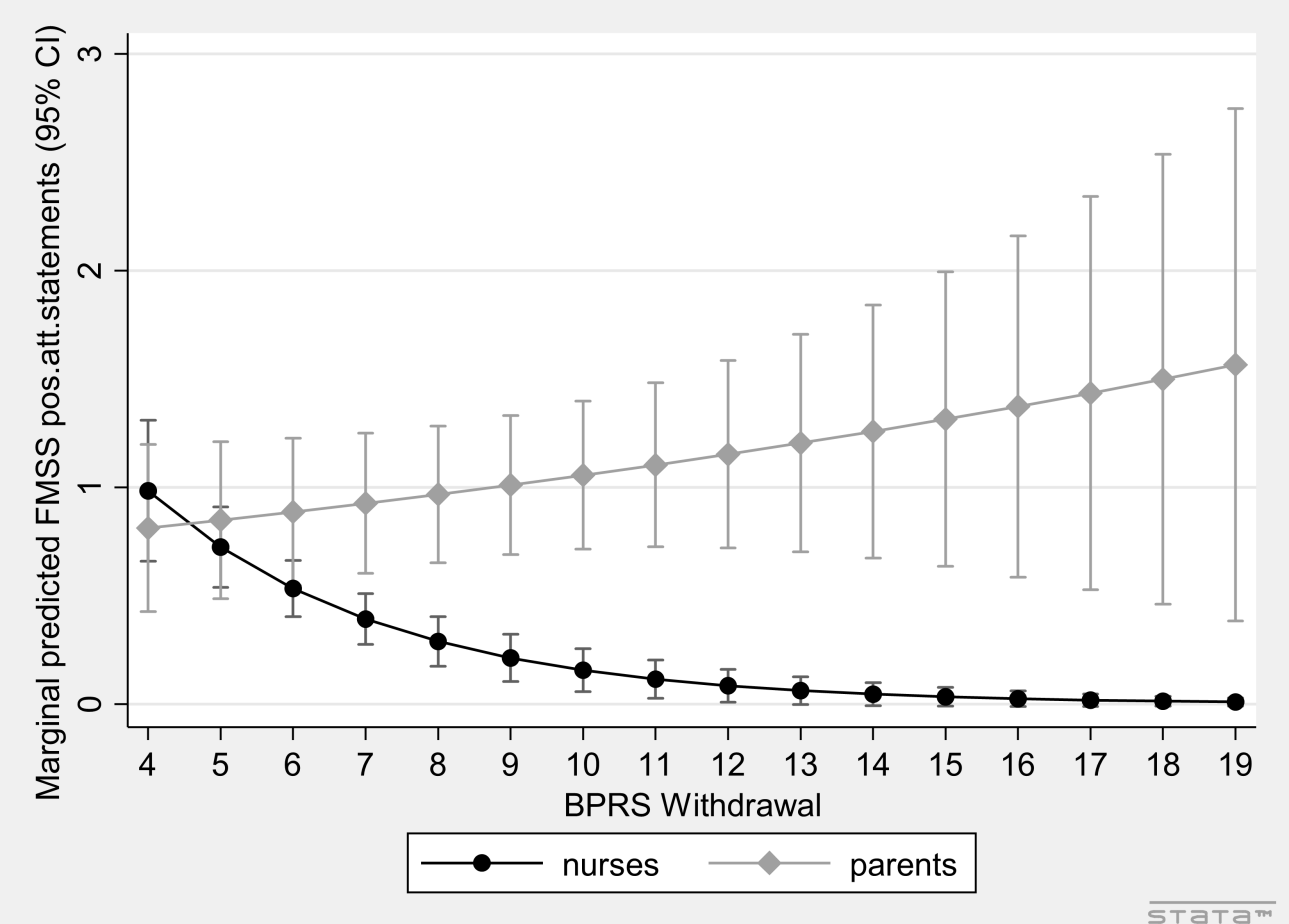
Effect of patients’ BPRS Withdrawal on FMSS positive attitude statements in the two settings (interaction plot).

In modified multivariate models for all EE outcomes in the total sample including patient group (outpatients vs. inpatients) and all patient-related predictors in stepwise models in either patient group (**Supplementary Table 5**), significant interaction with group was detected (one at a time) for BPRS Withdrawal on FMSS-EOI (interaction OR = 1.68, *p* = 5.3E-05) and for unemployed status (interaction IRR = 0.05, *p* = 0.005), pensioner status (interaction IRR = 0.02, *p* = 0.005), BPRS Withdrawal (interaction IRR = 1.42, *p* = 6.2E-06), and PC (interaction IRR = 1.23, *p* = 0.018) on FMSS positive attitude statements (**Supplementary Table 6** for simple slopes), suggesting that high EOI outcomes were associated with unemployed/pensioner status and negatively associated with BPRS Withdrawal and PC in inpatients only (**Figure 1**; **Supplementary Figures 1–3**). Only interactions of group with BPRS Withdrawal on both EOI outcomes survived our strict adjusted cutoff.

### Combining rater- and patient-related predictors of EE outcomes

In models combining rater- and patient-related predictors for EE outcomes, significance was lost for some predictors and gained for others. In inpatients (**Table 2A**), higher criticism was significantly predicted by higher nurses’ age, inpatients’ current employment and higher PC; higher EOI was significantly predicted by nurses’ higher age and shorter work experience, and inpatients’ higher age, lower disease duration, and lower BPRS Withdrawal and Hostility/Suspicion. In outpatients (**Table 2B**), higher criticism was significantly predicted by higher parents’ FBS Aggressive Behavior and outpatients’ higher education, being ever married, and history of suicide attempts; higher EOI was significantly predicted only by outpatients’ female gender. In combined models, no effect survived our strict adjusted cutoff, but the effect of inpatients’ BPRS Withdrawal on EOI survived our relaxed adjusted cutoff.

## Discussion

This study adds to a large literature on correlates of EE in families of individuals with schizophrenia as well as a smaller, more recent literature in staff–patient settings, e.g., community halfway houses (transitional hostels). Our study is novel in simultaneously recording EE across parents and professional caregivers. Most previous studies used patient–caregiver dyads after selecting one “primary” caregiver for each patient in order to simplify statistical analyses, yet unavoidably introducing bias. Instead, we have allowed each patient to be rated by one to two parents or two to five nurses. Therefore, another strength of this study was our recruitment scheme (“one patient by many raters, one rater for many patients”), which provided more valid and less biased EE ratings.

Previous meta-analyses of family EE in schizophrenia have reported a median high EE rate of 54% (range, 23%–77%) (28) and mean rates of 50.9% ± 12.5% (range, 23.3%–76.2%) for high EE, 46.9 ± 18.3% (range, 25.0%–94.1%) for high criticism, and 36.3% ± 17.6% (range, 12.0%–72.2%) for high EOI (10). Therefore, the rate of high EE in parents (**Table 1**) is at the upper end of meta-analytical reports but rates of high criticism and EOI are less than 1 SD higher than the means of previous studies in families. On the other hand, high EE rate in nurses was unexpectedly high, since rates are typically lower than 40% in staff–patient studies, with negligible rates of high EOI (14). Rates of high criticism were, expectedly, larger than those of high EOI in both settings. Cultural variation (29), individual characteristics of families and hostels, and author’s scoring style might provide explanation for these inflated EE rates.

Various caregiver- and patient-related predictors of criticism and EOI outcomes were identified in the two settings. **Table 3** provides a synopsis of findings in multivariate (**Tables 2A, B**) and univariate models (**Supplementary Tables 4A, B**). Several studies from a heterogeneous literature corroborated our findings; however, other studies were contradictory and various findings have, to the best of our knowledge, not previously been reported. High criticism in hostels was associated in previous studies with higher nurses’ age and longer work experience (30), lower education and lower personality openness (31), higher emotional burnout (32, 33), more time spent with patients (31, 34), causal attributions of disease to personal, internal, and controllable factors (35), and higher patients’ age and disease duration (31), poor job-related skills (30), patients’ overall psychopathology (particularly, negative symptoms/poor social functioning and aggressive/agitated behavior) (30, 31, 34–37), and higher PC (35, 38). In our study, high criticism in hostels was significantly predicted by higher nurses’ age and higher inpatients’ negative symptoms (univariate only) and PC, replicating previous findings, but also by inpatients’ current employment, not previously reported.

**Table 3:** Synopsis of findings (*p* < 0.1) for criticism and EOI in hostels (inpatients) and families (outpatients).

	Criticism						EOI					
	Hostels			Families			Hostels			Families		
	Uni	Multi	Comb Multi	Uni	Multi	Comb Multi	Uni	Multi	Comb Multi	Uni	Multi	Comb Multi
Caregiver predictors												
Female gender	–	NA	NA	-	NA	NA	**0.027**	**0.031**	0.064	0.086	**0.006**	-
Age	**0.048**	**0.048**	**0.008**	-	NA	NA	–	0.0002**	**0.018**	-	**0.009**	-
Nurse work experience (higher vs. lower)	–	NA	NA	NR	NR	NR	**0.049** **(−)**	5.6E-05** **(−)**	**0.003** **(−)**	NR	NR	NR
Nurse MBI emotional exhaustion	–	NA	NA	NR	NR	NR	–	0.093	–	NR	NR	NR
Nurse MBI personal achievements	–	NA	NA	NR	NR	NR	–	**0.036** **(−)**	–	NR	NR	NR
Parent education (high school vs. primary)	NR	NR	NR	0.090 (−)	NA	NA	NR	NR	NR	-	NA	NA
Parent FBS aggressive behavior	NR	NR	NR	**0.003**	**0.003**	**0.002**	NR	NR	NR	-	0.076 (−)	-
Parent FBS impact on activities/social life	NR	NR	NR	-	NA	NA	NR	NR	NR	-	**0.022**	0.093
Parent FBS impact on health	NR	NR	NR	-	NA	NA	NR	NR	NR	-	0.059 (−)	-
Parent FBS total	NR	NR	NR	0.067	NR	NR	NR	NR	NR	-	NR	NR
Patient predictors												
Female gender	–	–	0.051 (−)	-	NA	NA	0.066	0.097	–	-	**0.034**	**0.034**
Age	–	NA	NA	-	NA	NA	**0.004**	**0.002**	**0.008**	-	NA	NA
Family status (ever married vs. single)	–	NA	NA	0.076	**0.005**	**0.002**	–	NA	NA	-	NA	NA
Education (university or higher vs. lower)	–	NA	NA	-	-	**0.036**	–	NA	NA	0.064	NA	NA
Unemployed (vs. employed)	**0.007** **(−)**	**0.010** **(−)**	**0.010** **(−)**	-	NA	NA	**0.0014**	0.079	–	-	NA	NA
Pensioner (vs. employed)	**-**	**-**	**-**	-	NA	NA	**0.002**	0.051	–	-	NA	NA
Smoking	–	NA	NA	-	NA	NA	**0.039**	NA	NA	-	-	-
Disease duration	–	NA	NA	-	NA	NA	**0.027**	**0.036** **(−)**	**0.036** **(−)**	-	NA	NA
No.of previous hospitalizations	–	–	–	-	NA	NA	0.064	0.075	–	-	0.050 (−)	0.050 (−)
History of suicide attempts	–	NA	NA	-	0.052	**0.018**	–	**0.033**	–	-	NA	NA
BPRS thinking disorder	–	NA	NA	-	NA	NA	**0.020** **(−)**	NA	NA	-	NA	NA
BPRS withdrawal	**0.024**	–	–	-	0.060	-	4.9E-05** **(−)**	9.4E-06** **(−)**	0.00052* **(−)**	-	NA	NA
BPRS anxiety/depression	–	NA	NA	-	NA	NA	**0.026** **(−)**	NA	NA	-	NA	NA
BPRS hostility/suspicion	–	–	–	-	NA	NA	0.079 (−)	**0.011** **(−)**	**0.011** **(−)**	-	-	-
BPRS activity	0.093	NA	NA	-	NA	NA	**0.034** **(−)**	0.073	–	-	0.064	0.064
BPRS total	0.052	NR	NR	-	NR	NR	6.3E-05** **(−)**	NR	NR	-	NR	NR
Perceived criticism	**0.023**	**0.016**	**0.010**	-	NA	NA	**0.005** **(−)**	0.070 (−)	0.059 (−)	-	NA	NA

Uni, univariate models; multi, multivariate models with caregiver- or patient-related predictors only; comb multi, multivariate models with both caregiver- and patient-related predictors; hostels/inpatients, white columns; families/outpatients, gray columns.

For each predictor, the lowest p-value associated with any criticism outcome (FMSS-Criticism, number of critical comments) or any EOI outcome (FMSS-EOI, number of positive attitude statements) in uni, multi, and comb multi models is reported in each patient group.

p-values <0.1 are reported; -, p > 0.1; boldface values denote p < 0.05; ** p < 0.00022 (strict adjusted cutoff); * inpatients p < 0.00093, outpatients p < 0.00086 (relaxed adjusted cut-off); NA not included in model; NR, not relevant.

(−) negative effect (<1); otherwise, positive (>1).

BPRS, Brief Psychiatric Rating Scale; FBS, Family Burden Scale; FMSS, Five Minutes Speech Sample; MBI, Maslach Burnout Inventory.

High criticism in families was associated in earlier studies with parents’ reduced self-blame (39) and causal attributions of disease to personal, internal, and controllable factors (40, 41), overall caregiver burden and distress (39, 42–44), and patients’ male gender (45), both higher (46) and lower (42) patients’ age, unemployment (47), longer duration of untreated psychosis (43), more previous hospitalizations (47) or psychotic episodes (48), better cognitive functioning (47), higher PC (44, 49, 50), patients’ disturbed/aggressive behavior (51, 52), and higher depression/anxiety (47), but no other aspects of psychopathology (positive, negative symptoms) (39, 43, 53). We found that high criticism in families was significantly predicted by parents’ burden from their offspring’s aggressive behavior, corroborating previous findings, but also by outpatients ever being married, higher education, and history of suicide attempts (all three not previously reported).

High EOI in hostels was previously associated with higher staff neuroticism, more time spent with patients, and higher patients’ age (31). We found that high EOI in hostels was significantly predicted by nurses’ female gender, higher age, shorter work experience, and lower MBI Personal Achievements, and inpatients’ unemployed or pensioner status, smoking (both univariate only), lower disease duration (but higher in univariate models), history of suicide attempts, lower severity of psychopathology (i.e., negative symptoms/withdrawal and paranoia/hostility/aggression), and lower PC (univariate only), all of which were not previously reported, but also by higher inpatients’ age, replicating a previous report.

High EOI in families was earlier associated with relatives’ female gender (46, 51, 54, 55) but also male gender (42), unemployed status (55), higher caregiver burden and distress (39, 42–44), causal attributions of disease to universal, external, and uncontrollable factors (40, 41), less time spent with patient (42), and patients’ lower age (42, 46), unemployment (46), more previous hospitalizations (55), higher depression/anxiety, lower aggression (54), and higher PC (44). In this study, high EOI in families was significantly predicted by parents’ female gender and higher burden (specifically regarding social life), corroborating previous findings, but also by higher parents’ age and outpatients’ female gender, not previously reported.

The interpretation of our most robust findings might help unravel setting-specific pathogenetic pathways for EE components. After adjustment for multiple tests in models including either caregiver- or patient-related predictors, nurses displayed higher EOI when older or less experienced but lower EOI toward inpatients with more severe negative symptoms. FMSS-EOI is known to be positively correlated with CFI’s warmth dimension (i.e., concern for patients) through positive comments (7, 8). Therefore, younger and more experienced nurses (i.e., probably with higher levels of burnout) displayed lower interest in patients; furthermore, nurses showed less concern and were disengaged from individuals with more severe negative symptoms. This last effect survived in the final combined models and was the most robust finding in our study. It might be ascribed to causal attributions of negative symptoms to personal, internal, and controllable factors, such as personality weaknesses or laziness (35), or to low expectations of treatability and responsiveness to treatment. Importantly, the direction of causality between EE and patients’ behaviors cannot be inferred, and circular causation is highly probable. Interaction analyses identified our most robust differential EOI predictor; disengagement from individuals with higher levels of negative symptoms was recorded only in nurses. All aforementioned robust findings have not been previously reported. Several other caregiver- or patient-related features were nominally associated with criticism and EOI in each setting. However, no feature robustly predicted criticism in inpatients and criticism/EOI in outpatients after adjustment for multiple tests.

Our most robust findings might also help adjust the objectives or target groups of psychoeducational interventions to professional caregivers in supported housing facilities (56, 57), aiming to improve their caregiving capacity. Staff psychoeducation should aim to enhance concern for withdrawn patients, particularly of younger and more experienced nurses, by improving understanding of negative symptoms and modifying their causal attributions, by inspiring optimism about their responsiveness to therapeutic interventions, by improving nurses’ coping strategies toward negative symptoms, by motivating and supporting behavioral interventions specifically targeting negative symptoms and inactivity, and by engaging staff in vocational or social skills training programs for their patients (58, 59).

The limitations of our study include the following: (a) a relatively small sample size for both groups; (b) much fewer EE ratings in outpatients (56 in outpatients vs. 155 in inpatients), which restricted our power to detect significant associations in this group; although power analysis was not conducted before study initiation, we aimed at a twofold higher number of outpatients to compensate for less ratings in this group, but the COVID-19 pandemic outbreak thwarted further recruitment; (c) other patient (duration of untreated psychosis and cognitive functioning) or caregiver (time spent with patients, distress, causal attributions/illness perceptions, coping strategies, and personality profile) characteristics with previous evidence as EE predictors were not investigated in our study and should be included in future comparative studies.

In conclusion, we investigated patient- and caregiver-related predictors of EE toward individuals with schizophrenia living in halfway houses or with their families and explored differential effects across settings. Our most robust finding was that nurses displayed lower EOI toward individuals with more severe negative symptoms, i.e., they showed less concern and were disengaged from them. Interaction analyses showed that this effect was recorded only in nurses. Our findings might help unravel setting-specific pathogenetic pathways for EOI and customize psychoeducational interventions to the staff of supported housing facilities.

## Data availability statement

The raw data supporting the conclusions of this article will be made available by the authors, without undue reservation.

## Ethics statement

The studies involving humans were approved by “Attikon” University General Hospital. The studies were conducted in accordance with the local legislation and institutional requirements. The participants provided their written informed consent to participate in this study.

## Author contributions

PF: Conceptualization, Data curation, Formal analysis, Funding acquisition, Methodology, Software, Supervision, Validation, Writing – original draft, Writing – review & editing. SD: Data curation, Investigation, Project administration, Resources, Writing – review & editing. EK: Data curation, Formal analysis, Methodology, Software, Writing – review & editing. DD: Data curation, Investigation, Writing – review & editing. NS: Writing – review & editing. AD: Supervision, Writing – review & editing.
