# Supplementary material for: Differential predictors of expressed emotion toward individuals with schizophrenia between families and halfway houses
Source: Front Psychiatry. 2024 Mar 14;15:1322809. doi: 10.3389/fpsyt.2024.1322809 (PMC10973123; doi:10.3389/fpsyt.2024.1322809)

Suppl. Figure 1. Effect of patients’ BPRS Withdrawal on FMSS-EOI in the two settings (interaction plot).


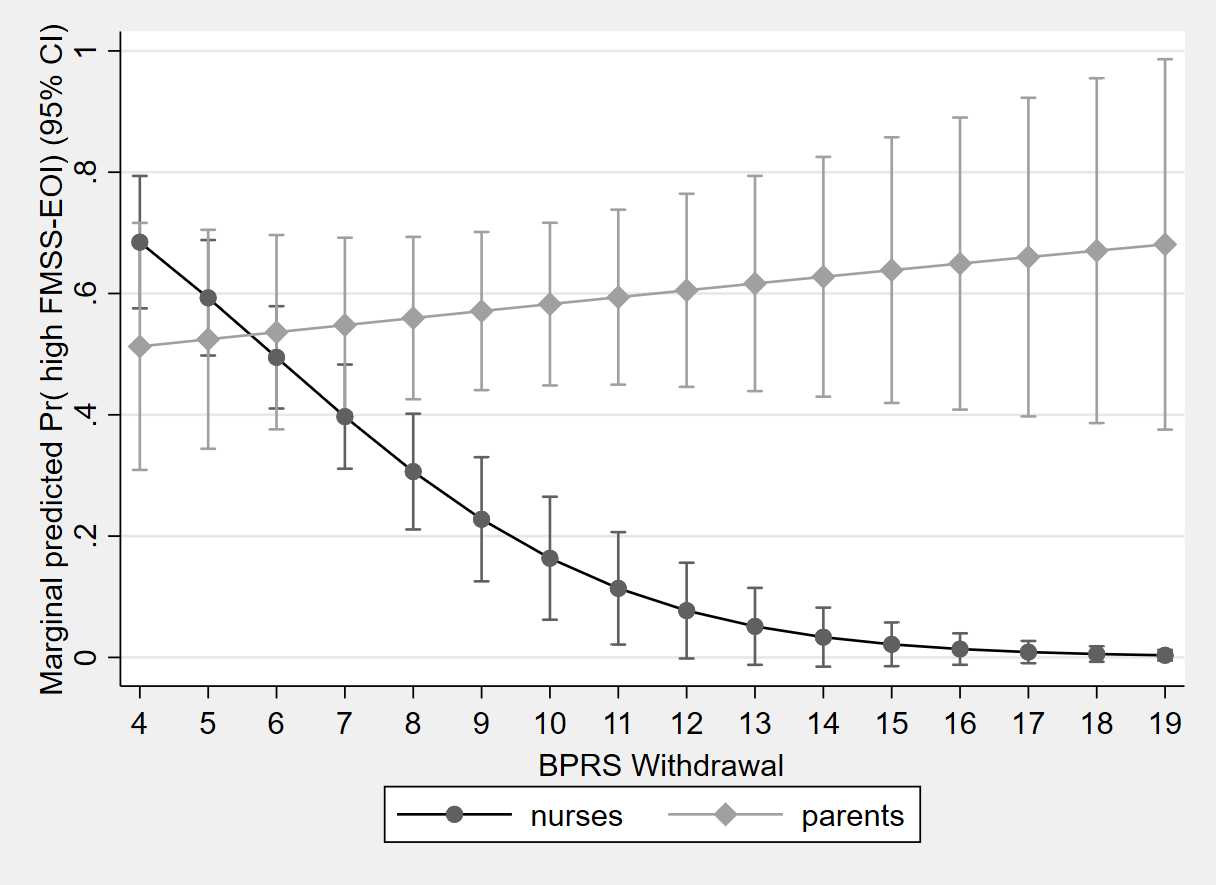


Suppl. Figure 2. Effect of patients’ employment status on FMSS positive attitude statements in the two settings (interaction plot).


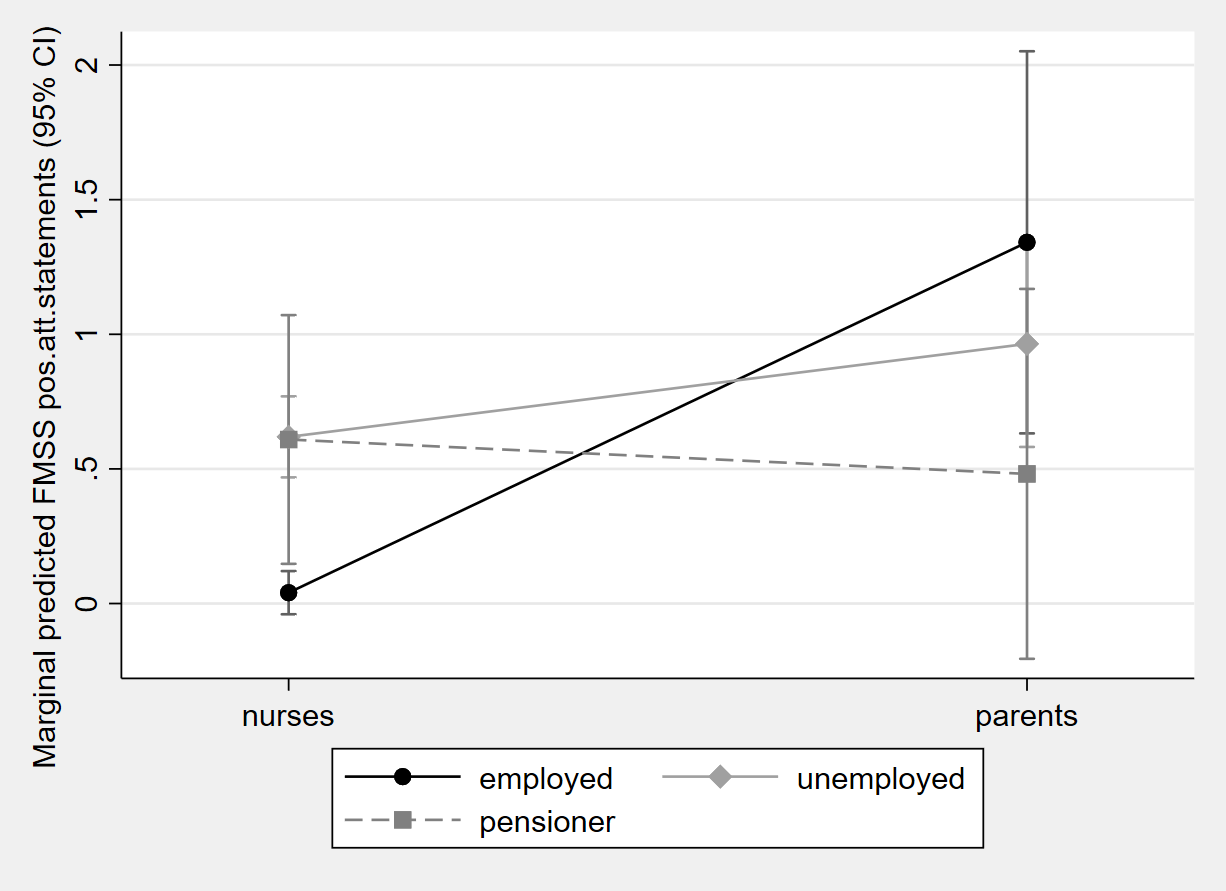


Suppl. Figure 3. Effect of patients’ Perceived Criticism on FMSS positive attitude statements in the two settings (interaction plot).


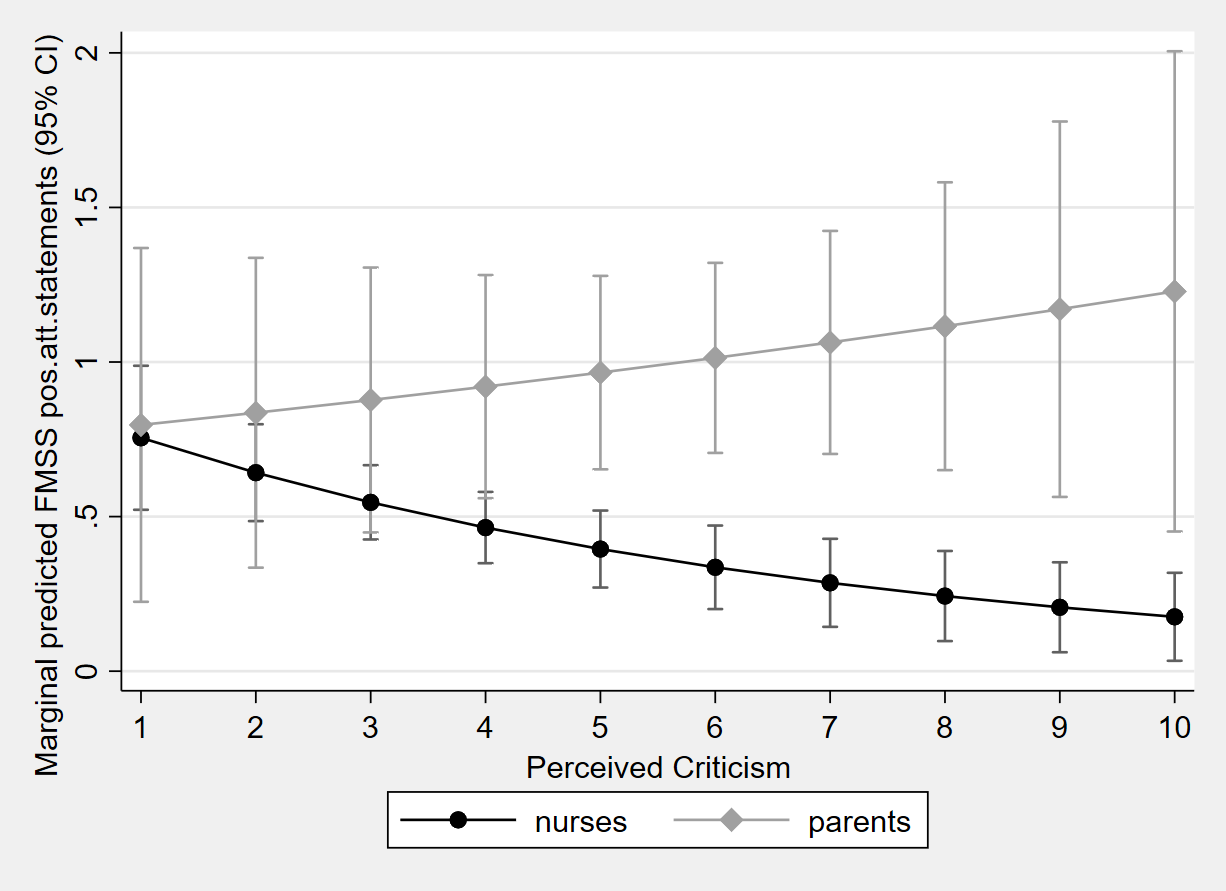

Supplement: Supplementary file 1 [file DataSheet_1.docx]
