# Supplementary material for: Differential predictors of expressed emotion toward individuals with schizophrenia between families and halfway houses
Source: Front Psychiatry. 2024 Mar 14;15:1322809. doi: 10.3389/fpsyt.2024.1322809 (PMC10973123; doi:10.3389/fpsyt.2024.1322809)
Supplement: Supplementary file 2 [file DataSheet_2.docx]

**Supplementary Methods**

**FMSS scoring**

Criticism and Emotional Overinvolvement (EOI), the two dimensions of EE, are rated based on conditions grouped in four categories: initial statement, quality of relationship, criticism, and EOI. The first three are used to rate Criticism and the last to rate EOI. Criticism and EOI are finally coded as high, borderline and low. Borderline can also be grouped with low to provide a dichotomous rating (high vs borderline/low).

**High Criticism** is coded if the rater (a) makes a negative initial statement about the patient; the initial statement can be rated as negative, neutral or positive; or (b) describes the relationship with the patient as negative; statements about the quality of the relationship are individually rated as weakly or strongly negative (-1 or -2) or positive (+1 or +2) and then summed up giving the overall relationship rating (negative, neutral or positive); or (c) makes at least one critical (i.e. strongly disapproving) comment, rated on the basis of content and voice tone. **Borderline Criticism** is coded if the rater only makes dissatisfaction comments.

**High EOI** is coded if the rater (a) reports or displays overprotective or self-sacrificing behavior or lack of objectivity towards the patient; or (b) exhibits intense emotional display during the interview; or (c) any two of the following are true: (1) describes the past or a minor characteristic of the patient in excessive detail, (2) makes at least one statement of positive attitude towards the patient, (3) makes five or more positive comments about the patient. **Borderline EOI** is coded if only one of the aforementioned (c) conditions is true.

The final seven EE categories arise from combining Criticism and EOI categories: ‘high critical’, ‘high EOI’, ‘high critical+EOI’, ‘borderline critical’, ‘borderline EOI’, ‘borderline critical+EOI’, ‘low critical+EOI’ (or ‘low EE’). If borderline is grouped with low, one will have four categories: ‘high critical’, ‘high EOI’, ‘high critical+EOI’, ‘borderline/low critical+EOI’. Finally, one can have two major categories of ‘high EE’ (critical and/or EOI) and ‘borderline/low EE’ (critical and/or EOI).

**Suppl. Table 1.** Patient sample Description (N=80).

|  | **Total**  **N=80**  **N(%)** | **Inpatients**  **N=40**  **N(%)** | **Outpatients**  **N=40**  **N(%)** | **p-value** |
| --- | --- | --- | --- | --- |
| **Gender** (Male) | 49(61.3%) | 27(67.5%) | 22(55.0%) | 0.251 |
| **Family status** |  |  |  | 0.965 |
| Single | 67(83.8%) | 34(85.0%) | 33(82.5%) |  |
| Married | 3(3.8%) | 1(2.5%) | 2(5.0%) |  |
| Divorced/Widowed | 10(12.5%) | 5(12.5%) | 5(12.5%) |  |
| **Education** |  |  |  | **0.002** |
| Primary/High School | 53(66.3%) | 33(82.5%) | 20(50.0%) |  |
| University or higher | 27(33.8%) | 7(17.5%) | 20(50.0%) |  |
| **Employment** |  |  |  | 0.709 |
| Employed | 17(21.3%) | 7(17.5%) | 10(25.0%) |  |
| Unemployed | 57(71.3%) | 30(75.0%) | 27(67.5%) |  |
| Pensioner | 6(7.5%) | 3(7.5%) | 3(7.5%) |  |
| **Smoking** | 52(65.0%) | 28(70.0%) | 24(60.0%) | 0.348 |
| **History of violent behavior** | 24(30.0%) | 14(35.0%) | 10(25.0%) | 0.329 |
| **History of suicide attempts** | 6(7.5%) | 3(7.5%) | 3(7.5%) | 0.999 |
|  |  |  |  |  |
|  | **Median(IQR)** | **Median(IQR)** | **Median(IQR)** |  |
| **Age (years)** | 43.5(38-50.5) | 49.5(42-56) | 40.5(37-44.5) | **0.001** |
| **Duration of disease (years)** | 15(7.5-22) | 18(9.5-27) | 13.5(7-19) | **0.047** |
| **No of hospitalizations** | 2(1-4) | 2(2-4) | 2(1-3) | **0.012** |
|  |  |  |  |  |
| **BPRS Thinking Disorder** | 6(4-8.5) | 5(4-7.5) | 6.5(5-9.5) | 0.190 |
| **BPRS Withdrawal** | 7(5-10) | 6(4-8) | 8.5(6-11.5) | **0.015** |
| **BPRS Anxiety/ Depression** | 8(6-11) | 7.5(6-9.5) | 9(6.5-12) | 0.162 |
| **BPRS Hostility/ Suspicion** | 4(3-6) | 4(3-5.5) | 4(3-6.5) | 0.964 |
| **BPRS Activity** | 3(3-4) | 3(3-4) | 3(3-4.5) | 0.345 |
| **BPRS Total** | 30(24-38.5) | 28(22.5-37) | 33.5(28.5-40.5) | **0.027** |
| **Perceived Criticism** | 5(2-6) | 4(1-5) | 6(5-8) | **0.001** |

N(%) or median(IQR) are presented. Chi-square, Fisher’s exact or Mann-Whitney tests were used as appropriate.

BPRS, Brief Psychiatric Rating Scale

Bold p<0.05

**Suppl. Table 2.** Questionnaire scores and reliability for patients (N=80), Nurses (N=22) and Parents (N=56).

|  | **No of Questions** | | **Range of Score** | | **Cronbach’s α** | | **Mean (SD)** | | **Median (IQR)** | |  |
| --- | --- | --- | --- | --- | --- | --- | --- | --- | --- | --- | --- |
| **Patients** |  | |  | |  | |  | |  | |  |
| **BPRS Thinking Disorder** | 4 | | 4-28 | | 0.728 | | 7.3(4.0) | | 6(4-8.5) | |  |
| **BPRS Withdrawal** | 4 | | 4-28 | | 0.703 | | 7.9(3.7) | | 7(5-10) | |  |
| **BPRS Anxiety/ Depression** | 4 | | 4-28 | | 0.726 | | 8.9(3.8) | | 8(6-11) | |  |
| **BPRS Hostility/ Suspicion** | 3 | | 3-21 | | 0.722 | | 4.7(2.1) | | 4(3-6) | |  |
| **BPRS Activity** | 3 | | 3-21 | | 0.712 | | 3.9(1.7) | | 3(3-4) | |  |
| **BPRS Total** | 18 | | 18-126 | | 0.783 | | 32.7(10.1) | | 30(24-38.5) | |  |
| **Perceived criticism** | 1 | | 1-10 | | - | | 4.8(2.7) | | 5(2-6) | |  |
|  |  | |  | |  | |  | |  | |  |
| **Nurses** |  | |  | |  | |  | |  | |  |
| **MBI Emotional Exhaustion** | 9 | | 0-54 | | 0.861 | | 12.4(8.5) | | 10.5(7-15) | |  |
| **MBI Personal Achievements** | 8 | | 0-48 | | 0.754 | | 37.3(5.3) | | 39(35-40) | |  |
| **MBI Depersonalization** | 5 | | 0-30 | | 0.703 | | 3.5(2.9) | | 3(1-5) | |  |
|  |  | |  | |  | |  | |  | |  |
| **Parents** |  | |  | |  | |  | |  | |  |
| **FBS Financial Burden (1)** | 5 | | 0-10 | | 0.701 | | 4.1(2.2) | | 4(2-6) | |  |
| **FBS Impact on Daily Activities/ Social Life (2)** | 8 | | 0-16 | | 0.796 | | 5.8(4.4) | | 5(2-8.5) | |  |
| **FBS Aggressive Behavior (3)** | 4 | | 0-8 | | 0.729 | | 1.0(1.4) | | 0.5(0-2) | |  |
| **FBS Impact on Health (4)** | 6 | | 0-12 | | 0.784 | | 5.2(3.4) | | 4(3-8) | |  |
| **FBS Total (1+2+3+4)** | 23 | | 0-46 | | 0.843 | | 16.1(8.6) | | 15(10-21.5) | |  |
| **FBS Objective Burden (1+2+3)** | 17 | | 0-34 | | 0.786 | | 10.9(6.3) | | 11(5.5-15.5) | |  |
|  | |  | |  | |  | |  | |  | |

BPRS, Brief Psychiatric Rating Scale; FBS, Family Burden Scale; MBI, Maslach Burnout Inventory

**Suppl. Table 3.** Rater samples description: Staff Nurses (N=22) and Parents (N=56).

| **Nurses (N=22)** | |  | **Parents (N=56)** | |  |
| --- | --- | --- | --- | --- | --- |
|  | **N(%)** |  |  | **N(%)** | **p-value** |
| **Gender** (Male) | 6(27.3%) |  | **Gender** (Male) | 24(42.9%) | 0.203 |
| **Education** |  |  | **Education** |  | **0.001** |
| Primary School | 0(0.0%) |  | Primary School | 20(35.7%) |  |
| High School | 8(36.4%) |  | High School | 19(33.9%) |  |
| University or higher | 14(63.6%) |  | University or higher | 17(30.4%) |  |
| **Work Experience** |  |  | **Employment** |  |  |
| 0-5 years | 3(13.6%) |  | Employed | 8(14.3%) |  |
| 5-11 years | 6(27.3%) |  | Unemployed | 11(19.6%) |  |
| >11 years | 13(59.1%) |  | Pensioner | 37(66.1%) |  |
| **Family Status** |  |  | **Psychiatric History** |  |  |
| Single | 8(36.4%) |  | No | 49(87.5%) |  |
| Married | 14(63.6%) |  | Yes | 7(12.5%) |  |
|  |  |  |  |  |  |
|  | **Mean (SD)** |  |  | **Mean(SD)** |  |
| **Age (years)** | 40.0(7.2) |  | **Age (years)** | 68.0(8.6) | **<0.001** |
|  |  |  |  |  |  |
| **MBI Emotional Exhaustion** | 12.4(8.5) |  | **FBS Financial Burden** | 4.1(2.2) |  |
| **MBI Personal Achievements** | 37.3(5.3) |  | **FBS Impact on Daily Activities/ Social Life** | 5.8(4.4) |  |
| **MBI Depersonalization** | 3.5(2.9) |  | **FBS Aggressive Behavior** | 1.0(1.4) |  |
|  |  |  | **FBS Impact on Health** | 5.2(3.4) |  |
|  |  |  | **FBS Total** | 16.1(8.6) |  |
|  |  |  |  |  |  |

N(%) or mean(SD) are presented. Chi-square, Fisher’s exact or Mann-Whitney tests were used as appropriate.

FBS, Family Burden Scale; MBI, Maslach Burnout Inventory

Bold p<0.05

**Suppl. Table 4a.** Univariate nurse- or parent-related predictors (p<0.1) of Expressed Emotion outcomes in inpatients and outpatients, respectively.

|  | **FMSS – Criticism**  **(OR, p)** | **FMSS –Critical comments**  **(IRR, p)** | **FMSS – EOI**  **(OR, p)** | **FMSS – Positive attitude statements**  **(IRR, p)** |
| --- | --- | --- | --- | --- |
| **Nurse predictors (inpatients)** | Logit, Nurses | NB, Nurses | Logit, Nurses | Poisson, Nurses |
| Gender (female vs. male) |  |  | **5.07, 0.027** |  |
| Age (years) | **1.05, 0.048** |  |  |  |
| Family Status (married vs. single) |  |  |  |  |
| Education (higher vs. secondary) |  |  |  |  |
| Work Experience (Ref. <5 years) |  |  |  | >11 y: **0.32, 0.049** |
| MBI Emotional Exhaustion |  |  |  |  |
| MBI Personal Achievements |  |  |  |  |
| MBI Depersonalization |  |  |  |  |
| **Parent predictors (outpatients)** | Logit, Patients | NB, Patients | Logit, Patients | Poisson, Patients |
| Relation/Gender (mother vs. father) |  |  | 3.11, 0.086 |  |
| Age (years) |  |  |  |  |
| Education (Ref. primary school) |  | High school:  0.33, 0.090 |  |  |
| Currently employed |  |  |  |  |
| Psychiatric History |  |  |  |  |
| FBS Financial Burden |  |  |  |  |
| FBS Impact on Activities/ Social Life |  |  |  |  |
| FBS Aggressive Behavior | 3.08, 0.051 | **1.90, 0.003** |  |  |
| FBS Impact on Health |  |  |  |  |
| FBS Total |  | 1.08, 0.067 |  |  |

FBS, Family Burden Scale; FMSS, Five Minutes Speech Sample; MBI, Maslach Burnout Inventory

Logit=Binary Logistic Generalized Linear Mixed Model; NB= Negative Binomial Generalized Linear Mixed Model; Poisson=Poisson Generalized Linear Mixed Model

Nurses= ratings were nested within nurses; Patients= ratings were nested within patients

OR>1 and IRR>1 denote positive associations.

Only predictors with p<0.1 are presented.

Bold, p<0.05

**Suppl. Table 4b.** Univariate patient-related predictors (p<0.1) of Expressed Emotion outcomes in the two patient groups.

|  | **FMSS – Criticism**  **(OR, p)** | **FMSS critical comments**  **(IRR, p)** | **FMSS – EOI**  **(OR, p)** | **FMSS positive attitude statements**  **(IRR, p)** |
| --- | --- | --- | --- | --- |
| **Patient Predictors** | Logit, Patients | NB, Patients | Logit, Patients | Poisson, Patients |
| Gender (female vs. male) | NA  NA | NA  NA | NA  NA | 1.92, 0.066  NA |
| Age (years) | NA  NA | NA  NA | **1.09, 0.004**  NA | 1.03, 0.082  NA |
| Family status (ever married vs. single) | NA  NA | NA  3.21, 0.076 | NA  NA | NA  NA |
| Education (university or higher vs. lower) | NA  NA | NA  NA | NA  2.78, 0.064 | NA  NA |
| Employment (Ref. employed) | Unemployed:  NA  NA  Pensioner:  NA  NA | Unemployed:  **0.35, 0.007**  NA  Pensioner:  NA  NA | Unemployed:  **24.62, 0.0014**  NA  Pensioner:  **71.04, 0.002**  NA | Unemployed:  **18.43, 0.005**  NA  Pensioner:  **21.16, 0.007**  NA |
| Smoking | NA  NA | NA  NA | **4.08, 0.039**  NA | NA  NA |
| Disease Duration | NA  NA | NA  NA | 1.05, 0.070  NA | **1.04, 0.027**  NA |
| No of previous hospitalizations | NA  NA | NA  NA | NA  NA | 1.10, 0.064  NA |
| History of violent behaviour | NA  NA | NA  NA | NA  NA | NA  NA |
| History of suicide attempts | NA  NA | NA  NA | NA  NA | NA  NA |
| BPRS Thinking disorder | NA  NA | NA  NA | **0.79, 0.020**  NA | NA  NA |
| BPRS Withdrawal | NA  NA | **1.18, 0.024**  NA | **0.64, 6.9E-05****  NA | **0.74, 4.9E-05****  NA |
| BPRS Anxiety/Depression | NA  NA | NA  NA | **0.83, 0.037**  NA | **0.88, 0.026**  NA |
| BPRS Hostility/Suspicion | NA  NA | NA  NA | NA  NA | 0.82, 0.079  NA |
| BPRS Activity | NA  NA | 1.26, 0.093  NA | **0.67, 0.034**  NA | 0.82, 0.097  NA |
| BPRS Total | NA  NA | 1.05, 0.052  NA | **0.88, 6.3E-05****  NA | **0.93, 0.0004***  NA |
| Perceived Criticism | **1.32, 0.023**  NA | NA  NA | **0.77, 0.035**  NA | **0.82, 0.005**  NA |

Upper line of each cell: inpatients; lower line of each cell: outpatients

BPRS, Brief Psychiatric Rating Scale; FMSS, Five Minutes Speech Sample

Logit=Binary Logistic Generalized Linear Mixed Model; NB= Negative Binomial Generalized Linear Mixed Model; Poisson=Poisson Generalized Linear Mixed Model; Patients= ratings were nested within patients

OR>1 and IRR>1 denote positive associations.

Only predictors with p<0.1 are presented; those with p≥0.1 are marked with NA.

Bold, p<0.05; ** p<0.00022 (strict adjusted cut-off); * inpatients p<0.00093, outpatients p<0.00086 (relaxed adjusted cut-off)

**Suppl. Table 5.** Modified multivariate models of Expressed Emotion outcomes in the total sample, including patient group and all predictors selected in multivariate stepwise models in either patient group (Tables 2a & 2b).

|  | **FMSS – Criticism**  **(OR, p)** | **FMSS critical comments**  **(IRR, p)** | **FMSS – EOI**  **(OR, p)** | **FMSS positive attitude statements**  **(IRR, p)** |
| --- | --- | --- | --- | --- |
| **Patient Predictors** | Logit, Patients | NB, Patients | Logit, Patients | Poisson, Patients |
| Group (outpatients vs inpatients) | 0.83, 0.724 | 0.47, 0.050 | **2.87, 0.017** | **2.34, 0.0001** |
| Gender (female vs male) | 0.69, 0.431 |  | 1.38, 0.382 | **1.61, 0.014** |
| Age (years) |  |  | **1.08, 0.004** |  |
| Family status (ever married vs single) |  | 1.45, 0.320 |  |  |
| Education (university or higher vs lower) |  | 0.89, 0.739 |  |  |
| Employment (Ref. employed) |  | Unemployed:  0.52, 0.067  Pensioner:  1.82, 0.274 |  | Unemployed #:  1.41, 0.232  Pensioner #:  1.08, 0.862 |
| Smoking |  |  |  | 1.28, 0.255 |
| Disease Duration |  |  | 0.97, 0.126 |  |
| No of previous hospitalizations | 1.04, 0.612 |  | 1.12, 0.099 | 1.03, 0.394 |
| History of violent behaviour |  |  |  |  |
| History of suicide attempts |  | 1.07, 0.893 |  | 1.21, 0.578 |
| BPRS Thinking disorder |  |  |  |  |
| BPRS Withdrawal | 1.05, 0.474 | 1.08, 0.056 | **0.83, 0.0006** # | 0.94, 0.063 # |
| BPRS Anxiety/Depression |  |  |  |  |
| BPRS Hostility/Suspicion | 1.08, 0.525 |  | **0.83, 0.045** | 0.90, 0.091 |
| BPRS Activity |  |  |  | 1.04, 0.524 |
| Perceived Criticism | **1.26, 0.018** | 1.11, 0.096 |  | 0.93, 0.079 # |

BPRS, Brief Psychiatric Rating Scale; FMSS, Five Minutes Speech Sample

Logit=Binary Logistic Generalized Linear Mixed Model; NB= Negative Binomial Generalized Linear Mixed Model; Poisson=Poisson Generalized Linear Mixed Model; Patients= ratings were nested within patients

OR>1 and IRR>1 denote positive associations.

# significant interaction with group

Bold, p<0.05

**Suppl. Table 6.** Interaction analyses of patient-related predictors with patient group (outpatients vs. inpatients) for Expressed Emotion (EE) outcomes.

| **EE outcome** | **Patient-related predictors** | **Interaction**  **(OR/IRR, p)** | **Simple slopes**  **(OR/IRR, p)** |
| --- | --- | --- | --- |
| FMSS-EOI | BPRS Withdrawal | **OR=1.68, p=5.3E-05** | inpatients OR=0.63, p=8.9E-05  outpatients OR=1.06, p=0.490 |
| FMSS positive attitude statements | Unemployed vs. employed | IRR=0.05, p=0.005 | inpatients IRR=15.35, p=0.008  outpatients IRR=0.72, p=0.301 |
| FMSS positive attitude statements | Pensioner vs. employed | IRR=0.02, p=0.005 | inpatients IRR=15.11, p=0.014  outpatients IRR=0.36, p=0.182 |
| FMSS positive attitude statements | BPRS Withdrawal | **IRR=1.42, p=6.2E-06** | inpatients IRR=0.74, p=1.0E-05  outpatients IRR=1.04, p=0.216 |
| FMSS positive attitude statements | Perceived Criticism | IRR=1.23, p=0.018 | inpatients IRR=0.85, p=0.004  outpatients IRR=1.05, p=0.482 |

BPRS, Brief Psychiatric Rating Scale; FMSS, Five Minutes Speech Sample

Bold, p<0.00022 (strict adjusted cut-off)
